# Web GIS in practice V: 3-D interactive and real-time mapping in Second Life

**DOI:** 10.1186/1476-072X-6-51

**Published:** 2007-11-27

**Authors:** Maged N Kamel Boulos, David Burden

**Affiliations:** 1 Faculty of Health and Social Work, University of Plymouth, Drake Circus, Plymouth, Devon, PL4 8AA, UK; 2 Daden Limited, 103 Oxford Rd, Moseley, Birmingham, B13 9SG, UK

## Abstract

This paper describes technologies from Daden Limited for geographically mapping and accessing live news stories/feeds, as well as other real-time, real-world data feeds (e.g., Google Earth KML feeds and GeoRSS feeds) in the 3-D virtual world of Second Life, by plotting and updating the corresponding Earth location points on a globe or some other suitable form (in-world), and further linking those points to relevant information and resources. This approach enables users to visualise, interact with, and even walk or fly through, the plotted data in 3-D. Users can also do the reverse: put pins on a map in the virtual world, and then view the data points on the Web in Google Maps or Google Earth. The technologies presented thus serve as a bridge between mirror worlds like Google Earth and virtual worlds like Second Life. We explore the geo-data display potential of virtual worlds and their likely convergence with mirror worlds in the context of the future 3-D Internet or Metaverse, and reflect on the potential of such technologies and their future possibilities, e.g. their use to develop emergency/public health virtual situation rooms to effectively manage emergencies and disasters in real time. The paper also covers some of the issues associated with these technologies, namely user interface accessibility and individual privacy.

## Background

When Google Earth and Google Maps first appeared many people marvelled at the ability to zoom in on almost any part of the planet and see objects at little more than 1 m resolution. However, the imagery is static, and relatively out of date. But what made Google Earth come alive was the ability to create so-called 'network links'–displays of data, often captured in real-time–which could be overlaid on the basic Google Earth mapping [1].

This capability is now (in November 2007) two years old. Whilst so-called 'mirror worlds' [2], such as Google Earth, have developed little further, the major innovation of the last 18 months has been the rise in popularity of 'virtual worlds', such as Second Life [3,4] (the US Department of Defense has been using virtual worlds since the 1990s). Here again it is the interfacing of the virtual space to real world data which can start to open up new possibilities in the ways that we view and analyse geographic data.

This paper describes some of the tools developed by Daden Limited [5] to explore the geo-data display potential of virtual and mirror worlds, and reflects on the potential of such technologies, their future possibilities, and some of the associated issues like user interface accessibility and individual privacy. Before looking at the tools in detail, it is worth putting these new technologies into context.

### The MetaVerse Roadmap and Metaverse 1.0 Consortium

A group of US companies and institutions active in this area recently published a Metaverse Roadmap [6]. This proposed that there were four emerging technologies that make up the so-called Metaverse–a digital domain equivalent to the atom based domain of our physical lives. These technologies are:

• *Mirror worlds*–digital representations of our own atom based world, such as Google Earth, Google Maps, and Microsoft Virtual Earth 3D;

• *Virtual worlds*–digital representations of any space, imagined or real, such as Second Life;

• *Lifelogging*–the digital capture of information about people and objects in the real (or digital) worlds; and

• *Augmented reality*–sensory overlays of digital information on the real (or even virtual) world, e.g., using head-up displays (HUDs).

Whilst there have been prototypes of systems in all four of these areas over the last 20 years or so (remember the Virtuality headsets of the 1980s), it is only in the last couple of years that these technologies have reached a maturity where they can be considered for serious use. Even then their adoption is likely to follow the order above, and useful, widespread deployment of some may yet still be a decade or so away.

The Metaverse 1.0 Consortium is a related group that includes over 40 participants of large and small/medium enterprises, as well as several research institutes and universities from eight participating countries [7]. Among the participants are IBM [8], Philips, Forthnet, Alcatel-Lucent, Telefonica I&D, Siemens IT, Barco, Geosim Systems Ltd., Technical University Eindhoven, Utrecht University, Technical University of Twente, Fraunhofer Rostock, Nazuka and Bertelsmann.

Metaverse 1.0 will provide a standardised global framework enabling the interoperability between various virtual and mirror worlds (virtual-virtual and virtual-mirror worlds interoperability) [9], and between them and the real world (sensors, actuators, vision and rendering, social and welfare systems, banking, insurance, travel, real estate and many others, enabling the realisation of 'mixed (real + virtual/mirror) reality' applications). The framework will be mainly driven by a set of selected application domains, including training, learning and simulation, eInclusion, and support for elderly, disabled and minorities, among other domains.

Within this paper we will primarily focus on mirror worlds and virtual worlds.

## Using mirror worlds and virtual worlds to display geographic datasets

### RSS and Google Earth

**Figure 1:**
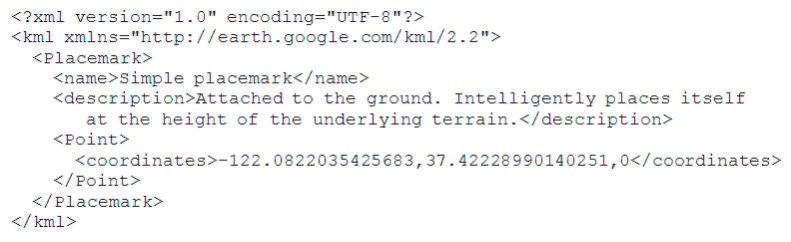
A simple KML file for Google Earth.

When we first encountered Google Earth in the summer of 2005 our attention was drawn to the network link facility. This lets you create a Keyhole Markup Language–KML [10] file (the eXtensible Markup Language–XML based standard used by Google Earth – Figure 1), place it on the Internet, and then have Google Earth users link their Google Earth browsers to it to display the information on their viewers [1]. At that time the majority of network link layers were static, and probably hand-crafted, KML files. However, there was some early work being done to capture live data (e.g., buses in California [11], or 911 calls in Seattle [12]) and to generate bespoke KML files automatically from them.

**Figure 2:**
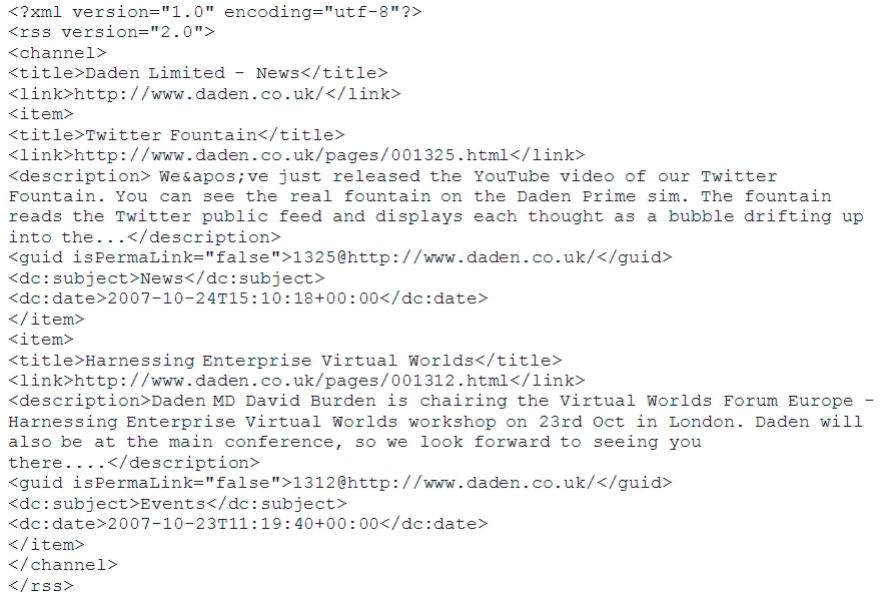
An example RSS feed.

We were interested in whether one could use more generic data sources than these. The most obvious choice was the RSS feed [13]. RSS is most commonly taken as standing for Really Simple Syndication. It is an XML based standard that can be automatically generated by many modern Content Management Systems (Figure 2) and lets Web sites (or other data owners) generate a list of updates to their Web site (or other data). Users can then subscribe to this feed by entering its URL (Uniform Resource Locator) into their own RSS reader or Web browser, and be alerted to any new content. In fact it is RSS that lies at the heart of podcasting.

The problem with using RSS feeds with Google Earth is that most such feeds do not contain geographically coded information. A case in point would be something like the BBC's World News RSS feed. To successfully plot such a feed onto Google Earth required us to develop a three stage process:

• Capture the feed;

• Parse it for geographic information, and geocode it; and

• Convert the data to KML.

#### Feed capture

Since RSS feeds are designed for public consumption by Web browsers they can be captured very simply and efficiently by a software programme that can make HTTP (Hypertext Transfer Protocol) requests out over the Internet. We do all our work in Perl [14] (seeing as most of our work is text rather than numbers or objects based), and Perl offers a library called LWP [15] to make this capture easy. The captured feed is just presented to the rest of the programme as a very long text string. Since we are dealing with pure text the capture time is often under a second–significantly less time than it takes a Web page to load.

#### Parsing and geocoding

This is the real challenge. From the simple text information in the feed we need to try and identify the geographic location of the item. For our work so far we have developed our own geocoder. This is a database of every country in the world, every major city, and every major airport, and the software searches the title (and optionally description) of the item for a place name it recognises. It then assigns the relevant geographic position for the item (*cf*. Metacarta's GeoParsing [16]). If a location is not found then the item is removed from the stream. We have also developed more detailed gazetteers for specific geographies (e.g., to village level in the UK), and it is possible to develop other bespoke gazetteers for specific clients and feeds. If data are already postcoded, then we can use postcode look-up services (such as Postcode Anywhere [17]) to convert from postcode to lat/long.

There are already some standards for geocoding RSS and similar data, such as ICBM [18] and GeoRSS [19]. Our application, which we call NewsGlobe [20], can identify when these formats are being used, so obviating the need for it to do its own geocoding.

#### Converting the data to KML

**Figure 3:**
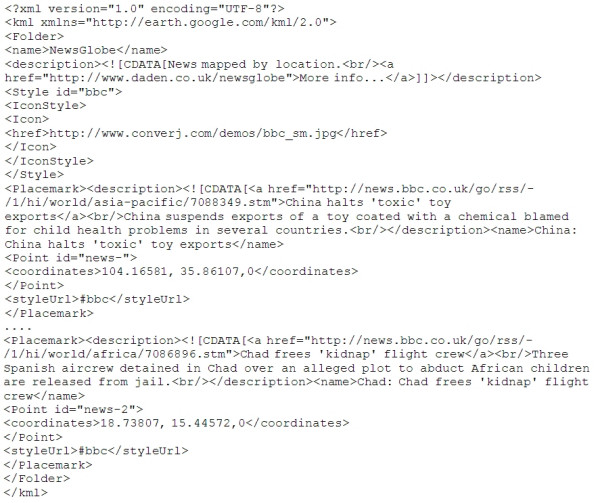
Automatically generated KML file from BBC News RSS feed.

The end result of the geocoding process is a 2-D array containing a record for each item, and fields for each of the required fields from the original data. NewsGlobe steps through this array and builds up the KML file (Figure 3).

### NewsGlobe in operation

**Figure 4:**
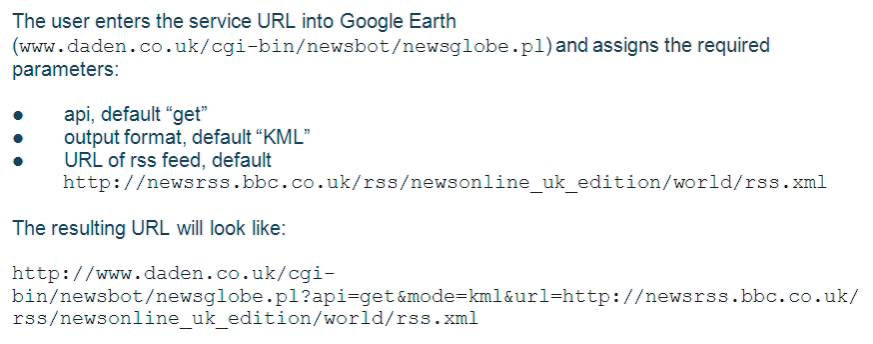
Accessing the NewsGlobe web service through a REST model interface.

**Figure 5:**
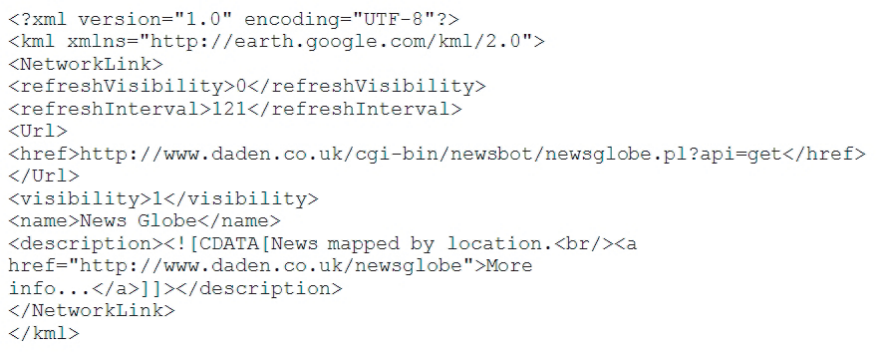
'Network link' file.

NewsGlobe, being a web service, is accessed through a REST (Representational State Transfer) model interface (Figure 4). Usually the resulting URL is wrapped within what is called a 'network link' in Google Earth, and this also offers the option to have the feed automatically refreshed on a timed basis (Figure 5).

When the feed is activated, Google Earth calls the NewsGlobe web service with the URL and parameters. NewsGlobe then makes its own HTTP GET request to the target feed URL, receives the RSS file, parses it as above, builds the KML file, and returns this file to Google Earth, which displays it. Unless otherwise specified, simple Google Earth markers are used. Each marker is assigned a label based on the <title> field for the item, and a pop-up description based on the <description> field of the item.

**Figure 6:**
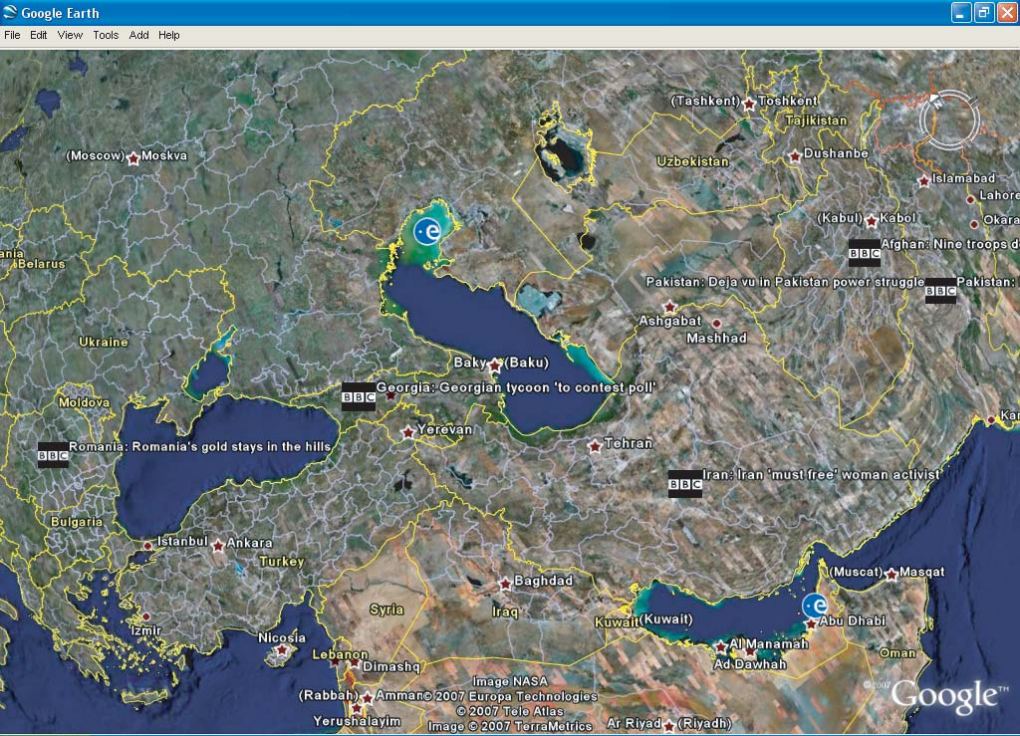
**NewsGlobe in action**. Google Earth displaying BBC News stories for 10 November 2007.

Figure 6 shows NewsGlobe in action, plotting stories from the BBC World News feed. Note that where two countries are mentioned in the story two markers have been created. Users can click on an icon to read the full news item given in the feed.

NewsGlobe was released and announced in July 2005. Within 3 months it was being used over a third of a million times a month. Usage has (thankfully) reduced since then, but it is still used by users every day to plot a range of news feeds.

### Google Maps and bird flu

**Figure 7:**
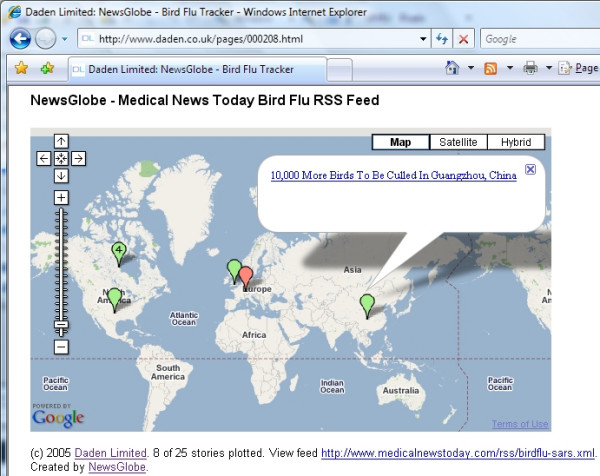
**Google Maps showing bird flu news**. Google Maps showing bird flu news stories for 11 November 2007 using NewsGlobe.

Having done NewsGlobe for Google Earth we then extended it to include Google Maps, primarily to avoid the need for all users to download Google Earth in order to view the geospatial data. Although the basic process is identical (and carried out by the same code), the end result is now a complete Web page (this was essentially pre-AJAX–the Asynchronous JavaScript and XML), with the Google Maps code and data embedded in it. The user requests this service by simply changing the API (Application Programming Interface) parameter to 'gmap'. At the time we were developing this map, bird flu was a major news item. So we identified a Web based RSS feed of avian flu news (in this case [21]), and used that as our example (Figure 7). Daden released the bird flu news map in December 2005, and it was picked up by media organisations and bloggers across the Web and is still accessed every day.

Since their development, Daden has continued to offer the NewsGlobe services for Google Maps and Google Earth for free on an as-is, non-commercial basis (details are at [20]). Readers are invited to make use of them for their own data.

### From mirror worlds to virtual worlds

Whilst Daden has been in Internet based virtual worlds since around 1996, and joined Second Life in 2004, it was not until Linden Lab (creator of Second Life) released the llHTTPRequest functionality [22] in the summer of 2006 that we felt that virtual worlds really had the opportunity to become serious business tools. Today one can find many RSS feed readers in various places around Second Life, as well as in-world scripted objects for posting blog entries from within Second Life (e.g., [23]), and a myriad of other objects that use LSL (Linden Scripting Language) HTTP Request and related functions to access the Internet and online databases outside Second Life. It was therefore fairly natural for us, when looking at ways to demonstrate the potential of these worlds, to go back to NewsGlobe and see whether we could achieve the same thing in a virtual world.

### DataGlobe

One of the major challenges of developing in Second Life is that its inbuilt scripting language (LSL) is very basic. Whilst it looks similar to JavaScript it suffers from major memory limitations (max 32 kb for program, data and working memory), a lack of shared libraries, enforced wait states, and a limit of 2048 bytes on any data returned by the llHTTPRequest call. Whilst this does limit what we can do in-world, by placing the majority of the programme on the Web we can produce useful applications. The LSL code essentially just manages the user interface, and accesses the application engine on the Web through its web service interface.

Visually DataGlobe is represented in Second Life by a 5 m tall globe showing a photographic whole-Earth image. This being a virtual world, one can instruct the globe to be bigger (to the 10 m limit of Second Life) or smaller (to a 1 m limit). One can also command it to rotate and tilt, and even change from photographic mapping to schematic mapping. If you users bored with the globe they can even tell it to morph into a 2-D map.

The operation is very similar to NewsGlobe, and again most of the code is re-used. As well as taking an ungeocoded RSS feed, DataGlobe can also take a KML feed – i.e., the data feeds used by Google Earth. This has the advantage that they are already geocoded. Instead of returning a KML file, NewsGlobe now returns a pipe-delimited text file, one line per record. Given the memory restrictions, the NewsGlobe API has been extended to allow the user to specify ways of limiting the amount of data returned to Second Life. For instance, title and description fields can be truncated to N characters, image links can be excluded, and lat/long can be rounded to integer or single decimal values.

**Figure 8:**
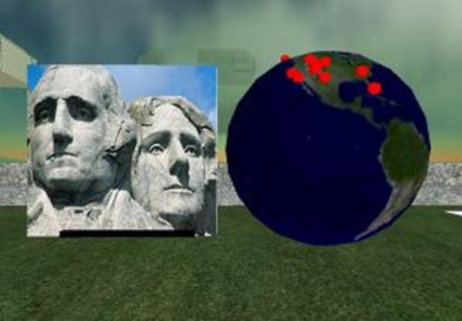
**DataGlobe in action**. DataGlobe [24] displaying the Discovery Channel KML Feed and Mt Rushmore image inside Second Life.

As with Google Earth though, the end result is a globe with markers (Figure 8[24]). Touching a marker will cause the marker to "say" (using Second Life text chat) its title and/or description. If a URL is associated with the item then this will be offered to the user (using a standard Second Life dialog, which will then let the user click through to the Web). If the item contains an image then it will be displayed (using Second Life's media parcel URL feature) on any nearby screen.

DataGlobe is available for free for non-commercial use. Please IM Corro Moseley (David Burden's avatar name) in Second Life for details.

**Figure 9:**
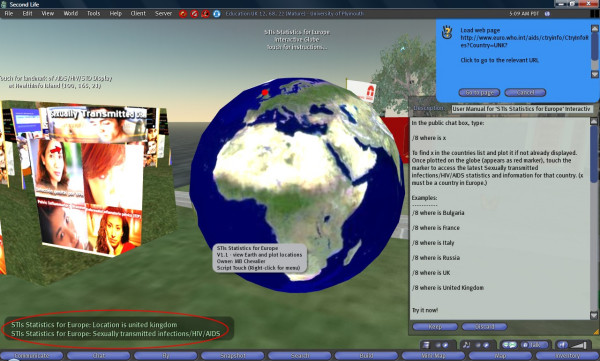
**Interactive 3-D Earth globe for accessing Web-based, geographically-indexed information**. An interactive 3-D Earth globe offering access to Web-based statistics and information about sexually transmitted infections (STIs)/HIV/AIDS from 53 European region countries (compiled by the WHO's Regional Office for Europe). The globe is part of the University of Plymouth Sexual Health SIM in Second Life [62]. Usage: In the public chat box, type: '/8 where is *country-name*' to find *country-name *in a list of 53 European region countries and plot it on the globe as red marker. Once plotted, touch the marker to access the latest STIs/HIV/AIDS statistics and information for that country on the WHO's Regional Office for Europe Web site.

As well as the NewsGlobe based version of DataGlobe, Daden also produced three other Second Life systems to explore mapping opportunities (available to view at [24]):

• A non-networked version of DataGlobe, where users can define the points to be plotted in a Notecard in Second Life (Figure 9). One can have more than one function/target information item associated all at the same time with a plotted location (Landmarks, Notecards, other Second Life Inventory items, and/or Web URLs). As well as Earth mapping, Daden also provides Mars and Moon images, and it should be possible to provide mapping graphics for other worlds or visualisations. A menu lets the user switch between different map points;

• A Second Life to Web mapping tool, where the Second Life user places markers out on a map and names them, and then touches the map to generate a Google Earth or Google Maps data feed, which can be viewed on the Web, i.e., the reverse of DataGlobe; and

• A UK map which shows weather by having intelligent "clouds", with each cloud fetching its own weather from the Yahoo! weather feed (*cf*. NOAA's 3-D real time USA weather data visualisation/map in Second Life [25]).

### GeoGlobe

One of the wonderful things about Second Life is that in many ways it works just like real life. Daden was working on the DataGlobe when its Second Life neighbour, Hayduke Ebisu (this is his Second Life name, and he is based in the USA), who is active in environmental issues in Second Life and real life, saw what the group was doing. He said that he had someone that Daden should meet. That person was Stephane Zugzwang (another Second Life name, and based in France). (Second Life is very much about social networking and collaboration [4]) Stephane had built a 'VR Room' in Second Life. This is similar to the 720 degree 'bubble photos' that are sometimes found on the Web, where one can pan and zoom in all directions. In the Second Life version, the photo is pasted onto the inside of a huge 20 m sphere, with the viewer's avatar standing inside and looking at the image all around her/him.

**Figure 10:**
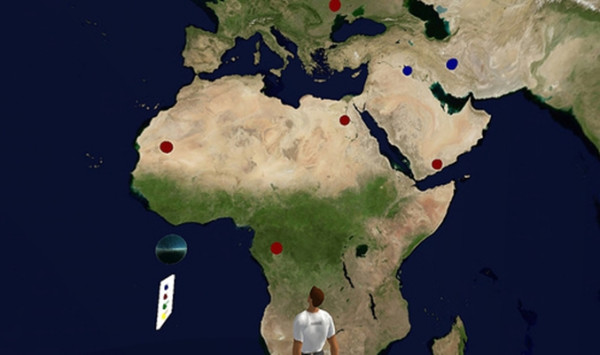
GeoGlobe in Second Life.

We all saw the potential to combine DataGlobe and VR Rooms. The resulting system was christened GeoGlobe (Figure 10[26]). GeoGlobe again shares the same NewsGlobe engine; all that changes is the final display UI (user interface). When a feed is selected the "points" of the feed fly out from a generator at the centre of the hollow sphere and "stick" to the map on the sphere wall at their correct locations. GeoGlobe also allows users to display multiple datasets, each in a different colour.

GeoGlobe received extensive blog coverage and can be seen at [27]. The technology has since been used by the Swedish Embassy in Second Life to display locations of Swedish Embassies around the world. Readers interested in using GeoGlobe for their own data are invited to contact Corro Moseley in Second Life.

### Real-time data

Whilst the NewsGlobe data were interesting, we still felt that there was more we could do in this area – particularly showing how real-time data could be used, and how we could move away from the 'pin-on-a-map' metaphor.

One of the Google Earth Dynamic Link Layers that had most impressed us had been one by US flight tracking company Fboweb.com [28]. Fboweb.com are official agents for the US Federal Aviation Administration's (FAA) Aircraft Situation Display to Industry (ASDI) [29] data. ASDI is a feed of all the radar tracks of aircraft around the USA, provided either in real time, or with a five-minute delay for security purposes. Fboweb.com used this data to produce a KML feed of the aircraft coming in to land at Los Angeles International Airport (LAX) [30]. One can zoom in on LAX and see the tracks of the aircraft, each identified with its flight number. Could we bring this feed into Second Life?

**Figure 11:**
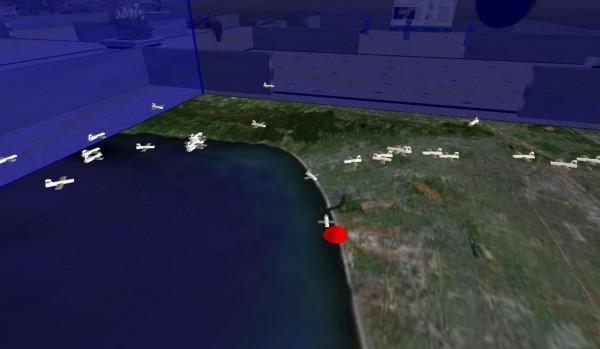
Daden's airplane tracking demo in Second Life.

First we have to unzip the Fboweb.com KMZ feed (KMZ is zipped KML to save space). We then extract the data for each aircraft, ignoring (at present) the historical track data and just extracting current aircraft location, altitude and heading. This is again put into a pipe-delimited format and returned to Second Life. In Second Life we have a 10 m × 10 m map of the relevant part of Southern California. Once the data arrive, the map creates a small model aircraft for each real aircraft, and places it at the correct scaled location and height above the map. The height scaling has been chosen so that cruising altitude is about head height to an avatar! With all the aircraft plotted the user can then walk through the data, walk up to an aircraft (which is labelled with its flight number), and touch it to gain additional information (flight route, speed and altitude). The display updates every minute, clearing the aircraft and plotting new ones (Figure 11). A video of the system is available on YouTube at [31]. The SLurl for the demo is at [32].

We did experiment with trying to animate aircraft between locations but at the 10 m map size we couldn't make them move slow enough – but it should be possible if a larger (100 m+) map was used (although this would also bring in new issues about how we 'rez' (resolve object in Second Life) the planes as there is a 10 m rez range limit, so we would need multiple 'aircraft generators'!).

The visualisation was launched at National Business Aviation Association (NBAA) Convention held in Atlanta, Georgia on 25–27^th ^September 2007, and has since been covered by both virtual world and aviation media.

Of course the important point about this demonstration is not the aircraft or the feed, but the way in which almost any sort of real-time or near real-time data (and even live GPS–Global Positioning System data feeds) can be visualised in ways in a virtual world that would be impossible in real life.

## Discussion

### Why virtual worlds?

**Figure 12:**
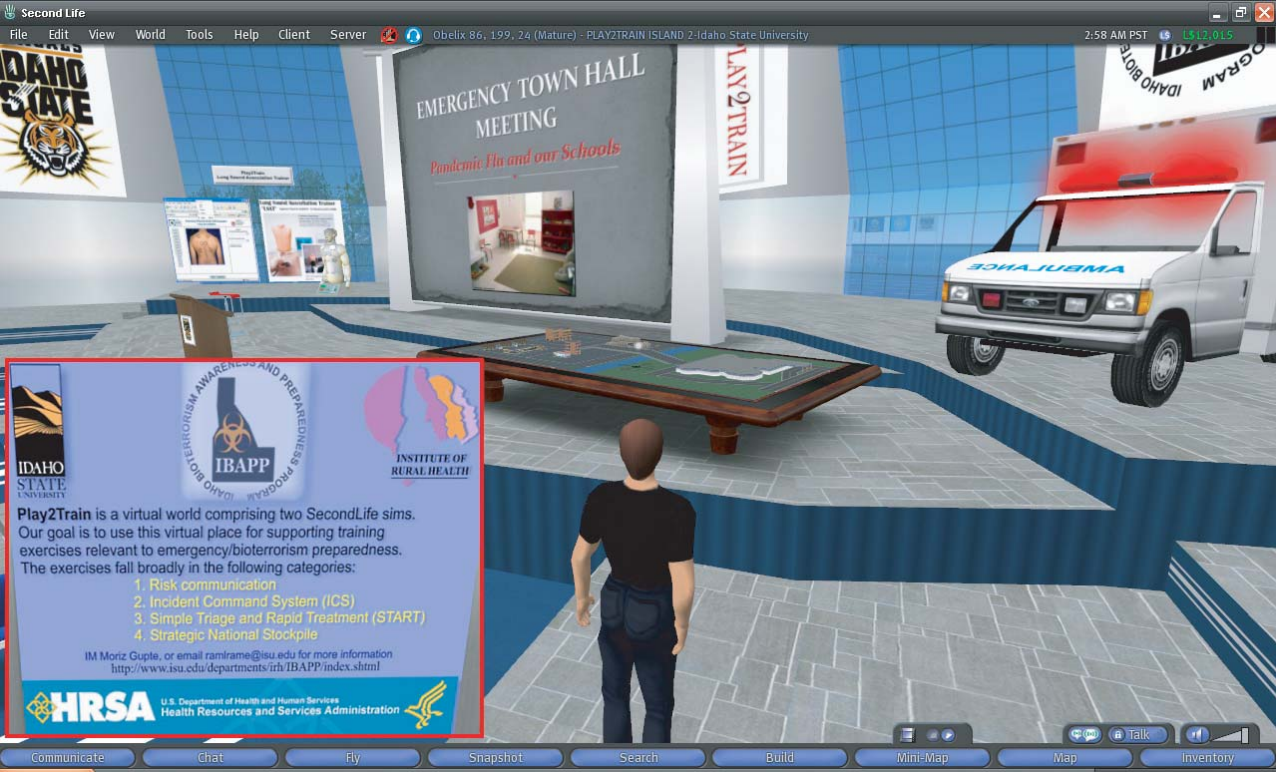
**Snapshot of Play2Train in Second Life**. Play2Train is a US federally-funded bioterrorism preparedness virtual training space in Second Life designed to support Strategic National Stockpile (SNS), Simple Triage Rapid Transportation (START), Risk Communication and Incident Command System (ICS) Training. This virtual environment spreads over two islands in Second Life, Asterix and Obelix (65,536 × 2 square metres and not open to public access), with one island dedicated to a virtual town and the other a virtual hospital. The design of this virtual environment is influenced by dioramas frequently used by emergency services to support their tabletop exercises [34]. Play2Train has been used in an Alternative Care Facility Mobile Quarantine and Healthcare Facility 'Sidewalk Triage' for a simulated avian flu pandemic. Similar technologies can be used to develop real-time emergency/public health situation rooms in the bridged mirror and virtual worlds, especially with the advent of virtual world features like sculpted prims, improved physics and 'HTML on a Prim' in Second Life [63] and application/desktop sharing in Sun's MPK-20 [64].

**Figure 13:**
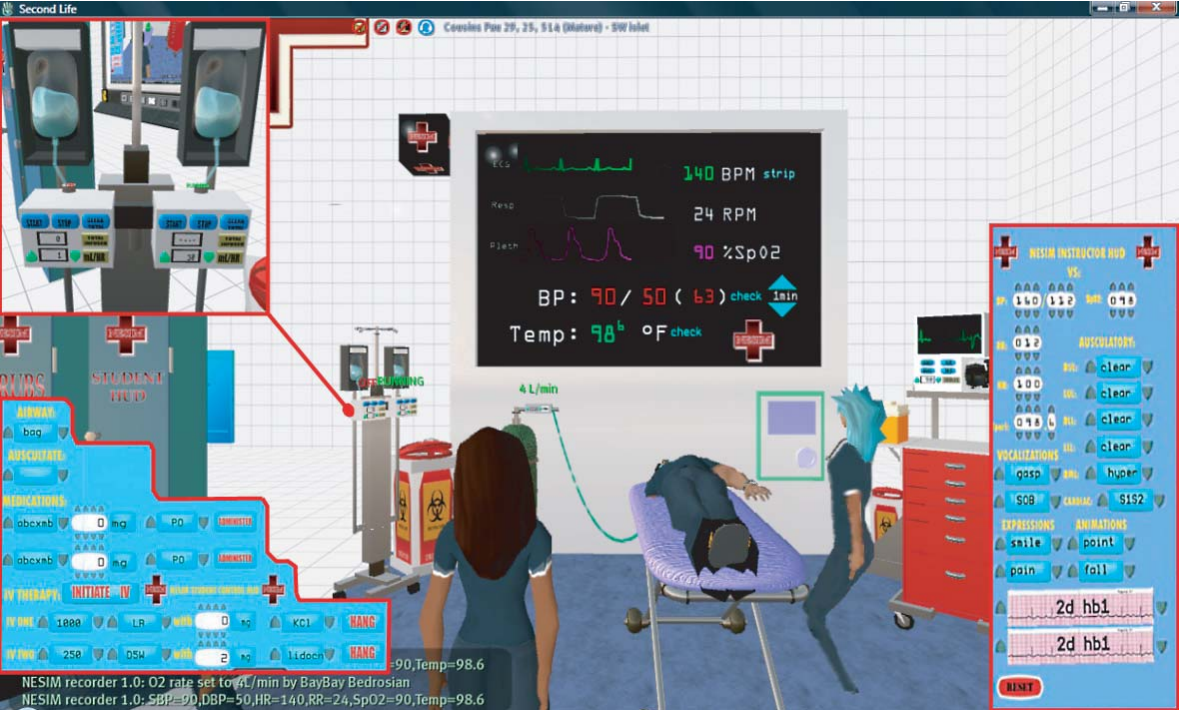
**Snapshot of NESIM in Second Life**. Snapshot of the Emergency/Nursing Education Simulator (NESIM) in action, showing both the student and instructor HUDs (insets). NESIM was developed by John Miller and colleagues at Tacoma Community College, WA, USA.

In truth almost any visualisation that we do in a virtual world could be achieved using a (probably bespoke or high power) desktop PC application. However, for us there are some undoubted advantages to doing such visualisations in a virtual world:

• *A single platform to learn and many uses*–one can use a single platform for the modelling and visualisation of a wide range of data, reducing the learning curve. Where else can one see both DNA molecules and civil aviation traffic being visualised at the same time?

• *Human behaviour modelling*–in applied epidemiology, a virtual world, being a social network, can be used as a unique disease modelling tool that incorporates important human behaviours for applied simulation modelling of infectious diseases [33];

• *Instant sharing*–the visualisation is instantly shareable with anyone who has a broadband connection and suitable PC anywhere on the globe, or it can be made private;

• *3-D simulations and real-time multi-user applications/virtual situation rooms*–the visualisation is not only passive, but can be interacted with and developed collaboratively – again on a global basis. An emergency/public health virtual situation room can be created in the virtual world, where avatars of experts and professionals, who might be in real life located in different geographic areas around the globe, can collaborate and discuss the data in real time, and even navigate together, and see, apply, and experiment with, changes to the simulated pseudo-physical space representing (again in real time) the real life location where the emergency/public health incident of interest is unfolding. The real-time link between the virtual world and the real world incident can be two-way and multimodal (involving sensor data feeds, textual exchanges, 3-D spatialised audio/voice, video feeds, 3-D simulations and animations, various Web mashups, shared desktop applications, etc.), which is useful for effectively managing the emergency situation in real time, rather than just watching it unfold. Pure (and cost-effective) simulations are also possible for training purposes; for example, Play2Train (Figure 12[34]) uses Second Life to create realistic virtual worlds and simulations for emergency and disaster preparedness training, while NESIM, the Emergency/Nursing Education Simulator (Figure 13[35]), enables live patient simulations, e.g., cardiac emergencies, to be created and role played by nursing students; and

• *A unique experience*–being able to walk or fly an avatar through the data gives us a more immediate and personal awareness of it, than being a third party viewer of the data. This may lead to unique insights about the data, which would be impossible or highly unlikely within a third person system.

Note that this latter renewed appreciation of data is more than just about subjective camera viewpoints (which can also be done with a desktop package). It appears to tap into a more primitive and natural appreciation of our surroundings, and the scale and location of the items in them relative to ourselves. From a psychological perspective, we "become" our avatar, experiencing things as the avatar sees them, rather than as a passive, real-world observer. We would love to see more research into this immersive nature of virtual world environments.

### 3-D modelling in mirror and virtual worlds

Despite its scripting limitations, Second Life remains a haven for content creators, as opposed to virtual worlds like There [36], where users cannot build anything completely of their own and are limited to choosing from some pre-determined sets of semi-customisable objects. However, some virtual worlds are starting to appear that can import 3-D models from Google's 3D Warehouse [37], a free and extensive library of 3-D models, something Second Life currently cannot do.

Google 3D Warehouse models can also be used in Google Earth. Google Earth uses the open COLLADA 3-D modelling format [38]. Using a Google program called SketchUp [39], users from around the world have built thousands of COLLADA models and made them freely available through Google 3D Warehouse. Microsoft's equivalent to Google's developments is their Virtual Earth mirror world with 3DVIA [40].

### On the convergence of mirror and virtual worlds, and the future 3-D Internet

In a recent article entitled 'Second Earth: The World Wide Web will soon be absorbed into the World Wide Sim: an environment combining elements of Second Life and Google Earth' and published in *MIT Technology Review *[41], Wade Roush discusses what will happen when Second Life and Google Earth, or services like them, actually meet. Roush rightly argues that "while Second Life and Google Earth are commonly mentioned as likely forebears of the Metaverse, no one thinks that Linden Lab and Google will be its lone rulers". What is coming is a larger digital environment combining elements of these technologies–the Metaverse or '3-D Internet'. Indeed, in January 2007, IBM predicted that the 3-D Internet will be one of the top five innovations that will change the way we live over the next five years [42].

The line between the real world and its virtual representations will soon start blurring [4,41]. The entire world is getting "wired" without wires: tiny radio and Internet-connected sensor chips are being attached these days to everything worth monitoring, including the human body [43,44]. But the real challenge is to organise and present the vast amounts of data these sensors generate in forms that diagnosticians and decision makers can make sense of. 'Reality mining' is the term that MIT Media Lab researchers and others are using for this emerging specialty [41]. And, as Roush [41] asserts, what better place (and metaphor) to mine reality collaboratively than in a social virtual space, where getting underneath, around, and inside data-rich representations of real-world objects is effortless?

### Individual privacy issues

Today mirror worlds like Google Earth present a real 'individual privacy challenge' by enabling everyone to see the full details of an individual's street/home, backyard and car on a 3-D colour map, along with corresponding online users' annotations and photos (thanks to GPS-enabled digital cameras), and even some relevant sounds [45] and YouTube videos [46]. Google Earth has become like a layered 3-D Wikipedia of the planet that anyone can edit and add to. It is becoming more and more easy to link an individual's home, work and the leisure/shopping and other locations they visit online or in the real world to other multimedia Web info about them and their family (compiled and published by them, their family, friends and colleagues, and others, in different contexts and at different times and places around the Web [47]), using the many Web 2.0/social networking information sources and mashup tools that are available today, e.g. Yahoo! Pipes [48] and Microsoft Popfly [49], among many others. These trends will only increase as 3-D geobrowsers like Google Earth become more and more integrated into 3-D social networks/3-D virtual worlds over the coming years. Indeed, "knowledge shall increase" (Daniel 12:4).

### Accessibility issues for users with special needs

People who are disabled in the real world sometimes face significant barriers to participation in 3-D mirror and virtual worlds like Second Life and Google Earth [50]. These worlds are essentially visually based environments, and as such the blind and visually impaired are excluded. However, researchers are currently working on spatialised audio interfaces for these worlds [51,52]. Voice communication in 3-D virtual worlds, which can be seen as an accessibility enhancer for some people having difficulties dealing with/communicating with typed text, is a challenge for the hard of hearing and deaf. But again technologies are being developed that automatically convert the spoken word to Sign Language using speech recognition to animate an avatar [53]. Easy, smooth movement through 3-D mirror and virtual worlds requires more fine motor control than what some disabled users possess. Motion-sensitive controllers that use multi-axial accelerometer-based sensors like the Wiimote [4] and enhanced 3-D mouse navigation [54] could offer hope here. Many disability barriers can also be overcome through individualised coaching and mentoring support provided to new users with special needs to help them have a better 3-D mirror and virtual world experience, including special registration, orientation and support portals designed for this purpose (both out-world on the flat Web and in-world).

Location-aware mobile device interfaces to the future Metaverse [55-57] will also need to carefully designed to make them accessible and usable by not just the disabled, but also the non-disabled, given the smaller screen sizes of these devices, their special input modalities, their intended applications/uses (e.g., in mobile augmented reality), and other particularities.

## Conclusion: Geography 2.0 and the democratisation of GIS

To us this whole journey from Google Earth to virtual worlds like Second Life has been about the democratisation of GIS (Geographic Information Systems), so that they are no longer only associated with big proprietary names and solutions. New technologies like those described in this paper bring Web 2.0 approaches to GIS [58], and indeed this whole area has been referred to as Geography 2.0 [59]. They let almost anyone with a modicum of programming skills mash-up varying data sources with equally varying display modes to create unique visualisations of data, and then to combine those mashups with yet others to create ever more fascinating and useful images of the datasphere.

And this is just the start. Technologies such as Yahoo! Pipes [48] will let non-programmers create mashups and novel visualisations without the need to programme, combining data feeds and filters with almost Lego-like simplicity. MIT's SIMILE project [60], for example, has created Timeline, a kind of "Google Maps" for time series data, whether one is tracking eons, milliseconds, or both. And in the next few years we are bound to see a collision between the Metaverse technologies of mirror worlds, virtual worlds, augmented reality and lifelogging, and be able to capture, visualise and analyse data in ways that only a decade ago we could only dream of.

Interested readers are further referred to the online section entitled 'Second Life GIS' at [61], which features many news and pointers that are directly related to the topic of this paper.

## Competing interests

DB is Managing Director of Daden Limited, an Information 2.0 consultancy and full service Virtual Worlds/Second Life development agency.

## Authors' contributions

MNKB and DB contributed equally to the manuscript. DB provided insider information about Daden's technologies and tools presented in this paper. Both authors read and approved the final manuscript.
